# Synergistic anticancer effect of ellagic acid and Kaempferol combination in a mouse model of breast cancer

**DOI:** 10.3389/fonc.2026.1754560

**Published:** 2026-05-28

**Authors:** Rand Jabbar Shihab, Moudi M Alasmari, Heba K Alshaeri, Márta Hock, Wamidh H. Talib, Sergej Ostojic

**Affiliations:** 1 Department of Clinical Pharmacy and Therapeutics, Faculty of Pharmacy, Applied Science Private University, Amman, Jordan; 2 College of Medicine, King Saud bin Abdulaziz University for Health Sciences (KSAU-HS), Jeddah, Saudi Arabia; 3 King Abdullah International Medical Research Centre (KAIMRC), Jeddah, Saudi Arabia; 4 Department of Pharmacology, Faculty of Medicine, King Abdulaziz University, Rabigh, Saudi Arabia; 5 Institute of Physiotherapy and Sports Science, Faculty of Health Sciences, University of Pécs, Pécs, Hungary; 6 Physical Activity Research Group, János Szentágothai Research Center, University of Pécs, Pécs, Hungary; 7 Faculty of Allied Medical Sciences, Applied Science Private University, Amman, Jordan

**Keywords:** anticancer therapies, breast cancer, combination treatment, ellagic acid, Kaempferol

## Abstract

**Background:**

Cancer continues to be a major global health issue and is one of the leading causes of death. Breast cancer ranks among the most common neoplasms worldwide. Natural products have shown notable advantages in cancer treatment. Ellagic acid (EA), a polyphenolic compound found in natural sources, has potent anticancer properties. Kaempferol (KAE) is a natural substance known for its strong anticancer activity. This study investigated the antitumor activity of the combined use of EA and KAE on various cancer cell lines.

**Methods:**

*In vitro*, EA, KAE, and their combination were evaluated for their antiproliferative activity against five cancer cell lines: MDA-MB-231, T47-D, HeLa, EMT-6/P, A549, as well as the non-cancerous Vero cell line. The impact of each treatment on cell viability was examined using the MTT assay. T47-D cells were treated with EA, KAE, and a combination of both, and caspase-3 activity was measured. For the *in vivo* study, EMT-6/P cells were injected into *Balb/C* mice, which were treated with EA, KAE, and their combination. Tumor size and weight were measured, and hepatotoxicity and nephrotoxicity were also investigated for all treatments.

**Results:**

*In vitro*, the combination of EA and KAE exerted a synergistic antiproliferative effect across all tested cancer cell lines, with the most pronounced effect observed in T47-D cells. This combination produced low toxicity in the non-cancerous Vero cell line. Furthermore, the combination led to a greater induction of apoptosis compared to single-agent treatment. *In vivo*, the EA and KAE combination exhibited a notable reduction in tumor volume (p< 0.05) and increased proportion of tumor-free mice. Safety evaluations showed no signs of liver or kidney toxicity in the combination-treated group (p > 0.05).

**Conclusion:**

The combination of EA and KAE represents a promising candidate for breast cancer treatment due to its synergistic effect. It maximizes therapeutic potential through the induction of apoptosis.

## Introduction

Breast cancer is the most prevalent cancer among women globally and remains a leading cause of cancer-related fatalities in this population (1). Its management is particularly challenging due to its high metastatic potential and the frequent development of resistance to conventional chemotherapeutic agents (2). Projections indicate that the breast cancer incidence could rise by over 40% by 2040, potentially reaching three million new cases each year. Likewise, mortality rates are anticipated to increase by more than 50%, with annual deaths potentially reaching one million by the same year (3). According to available statistics, breast cancer has a major impact on societies worldwide, and there remains an urgent need for effective treatments (4). Multidrug resistance (MDR) further complicates conventional therapies by weakening their efficacy (5). The harsh adverse effects and diminished quality of life associated with these treatments emphasize the need for more tolerable therapeutic alternatives.

Natural products are defined as compounds found in nature with a range of biological activities and selective targeting of cancer cells over normal cells (6). They can be obtained from diverse sources, including plants, bacteria, and marine organisms (7). Natural products have shown notable advantages in cancer treatment, such as potent antitumor activity and minimal toxicity (8). They also exhibit lower cellular resistance, reduced costs, and offer valuable approaches for the development of new drugs (6). Notably, over 83% of chemotherapeutic agents approved by the Food and Drug Administration (FDA) are derived from natural sources (9).

Ellagic acid (EA) and Kaempferol (KAE) stand out among natural products for their extensively researched anticancer prowess. EA is a naturally derived polyphenol found in various fruits, nuts, and vegetables (10). EA has attracted growing interest due to its wide range of biological activities and potential health benefits. It is especially known for its strong antioxidant properties (11), anti-inflammatory effects (12), antibacterial activity (13), as well as cardioprotective (14), neuroprotective effects (15). Regarding its anticancer activity, EA has been shown to induce apoptosis and reduce the proliferation of breast cancer cells, partly by targeting Cyclin-Dependent Kinase 6 (CDK6) (16). KAE, also known as indigo yellow, is another plant-based compound with notable health benefits. It is found in a variety of fruits and vegetables, including berberis, citrus fruits, broccoli, grapes, and tea leaves. The therapeutic properties of KAE, particularly its antioxidant effects, are linked to the phenolic compounds in its chemical structure (17). KAE may influence cancer growth and progression through its antioxidant and anti-inflammatory properties by restoring redox balance (18). In addition, KAE demonstrates significant antiproliferative effects on various types of breast cancer cells by impairing mitochondrial function and inducing apoptosis (19). Although EA and KAE are both classified as polyphenolic compounds and share antioxidant properties, available evidence indicates differences in their reported molecular targets. EA has been linked more prominently to cell cycle regulation, including CDK6-related signaling, whereas KAE has been associated with mitochondrial-mediated apoptosis and pathways such as PI3K/AKT. On this basis, the combination of EA and KAE was examined to determine whether concurrent modulation of cell cycle progression and mitochondrial apoptotic signaling could enhance antitumor activity compared with single-agent treatment. While combinations of polyphenols have been investigated in cancer research, the specific interaction between EA and KAE has not been evaluated in breast cancer models using both *in vitro* and *in vivo* approaches.

## Methods

### Chemicals, cell lines and cell culturing condition

Dulbecco’s Modified Eagle Medium (DMEM), Minimum Essential Medium (MEM) and Roswell Park Memorial Institute (RPMI-1640) were obtained from (Sigma, USA). Culture media were supplemented with 0.1% gentamycin, 1% penicillin-streptomycin solution, 10% fetal bovine serum, and 1% L-glutamine (Sigma, USA). Trypsin ethylene diamine tetra acetic acid (trypsin-EDTA) and phosphate buffer saline (PBS) (Sigma, USA), were the two reagents used in the trypsinization procedure. Trypan blue solution (0.4%; Sigma, USA) was used for cell counting and viability assessment. Dimethyl sulfoxide (DMSO; Alpha Chemika, India) was used for dissolving EA and KAE and as a solvent in the MTT assay.

EA and KAE were procured from (Sigma, USA) as certified analytical standards with a purity ≥98%. These compounds were used as received without further purification. Cytotoxicity was evaluated using six cell lines: MDA-MB-231, T47-D, HeLa, EMT-6/P, A549 and Vero. The cell lines present in this study were obtained from the American Tissue Culture Collection (ATCC). Cells were cultivated in complete culture media and kept in an incubator with 5% CO2 and 95% humidity at 37°C. MDA-M B-231 and HeLa and A549 cells were cultured in DMEM media, T47-D cells were cultured in RPMI-1640 medium (20), while Vero and EMT-6/P cells were cultured in MEM media (21).

### Analysis kits

MTT (3-(4,5-Dimethylthiazol-2-yl)-2,5-diphenyltetrazolium bromide) assay kit (Bioworld, UK) was utilized to evaluate antiproliferative activity. Alanine aminotransferase ALAT (GPT) FS kit (BioMajesty^®^, Germany). Aspartate aminotransferase ASAT(GOT) FS kit (BioMajesty^®^, Germany). Creatinine FS assay kit (BioMajesty^®^, Germany) were used to evaluate liver and kidney function. Human Caspase-3 ELISA Kit (Sunlong Biotech, china) With a Catalogue Number: SL2079Hu was used to determine apoptotic cell response.

### Preparation of ellagic acid and kaempferol working solutions

To prepare treatments for the MTT assay, EA 10 mg was dissolved in 0.5 mL DMSO to obtain a stock solution of 20 mg/mL (66 mM), while KAE,28.5 mg was dissolved in 1 mL DMSO to prepare a stock solution of 28.5 mg/mL (100 mM). Further dilutions were prepared from these stock solutions. For working solutions, 1.5 μL of EA stock solution and 1 μL of KAE stock solution were diluted with complete tissue culture medium to a final volume of 1 mL, yielding a final concentration of 100 μM for each compound in single-treatment experiments. Serial dilutions were subsequently prepared by reducing the concentration by 50% at each step.

In all experiments, the final DMSO concentration in the treatment media was kept below 0.25% (v/v), a concentration that does not influence cell viability.

### Anti-proliferative (MTT) assay

After the selected cell line was cultured, trypsinized and its cells counted, a multichannel pipette was used to seed the cells at a concentration of 15000 cells per well into a 96-well tissue culture plate (100μl/well). The plate was incubated for 24 hours to ensure appropriate cell adhesion and growth. Following incubation, the adherent cells were treated in triplicate with decreasing concentration of EA and KAE (100,50,25,12.5,6.25,3.12,1.56,0.78 μM) for single treatments and combination treatments consisted of decreasing concentrations of one compound in the presence of a fixed concentration of the other, resulting in a total volume of 200 μl/well, after the complete emptying of the medium for every well. Following incubation for 48 hours, cell viability was measured using the MTT (the tetrazolium salt, 3, [4,5-dimethylthiazol-2- yl]-2,5-diphenyltetrazolium bromide) assay kit (Bioworld, UK). The primary goal of the experiment was to ascertain how mitochondrial dehydrogenase reduces MTT to produce blue formazan crystals, which indicate viable mitochondria and cell survival. The procedure was performed as follows: the treatment-containing medium was removed from each well, and the cells were gently washed with PBS. Next, 100 μl of culture media and 10 μl of MTT solution was added to each well. After incubating for three hours, 100 μl of DMSO was added to the plates. Adding DMSO serves to dissolve the formazan particles that had developed in the cells that were still alive.

The plate was incubated for one hour, and then the optical density (OD) at 550 nm was measured using a microplate reader. Cell viability (% survival) was calculated for all treatments and compared with the negative control cells (untreated cells maintained in culture medium). The percentage of surviving cells was calculated using Microsoft Excel software.


$$\text{Percentage of Cell Viability}\left(\%\right)=\frac{\text{OD of treated cell}}{\text{OD of control cell}}*100$$

### Calculation of the half maximal inhibitory concentration IC_50_

The IC_50_ represents the concentration of a drug required to achieve 50% inhibition or cell death compared to untreated cells, typically determined using molar concentration. In this study, IC_50_ values for single and combination treatments were calculated using nonlinear regression analysis in the Statistical Package for the Social Sciences (SPSS).

### Calculation of combination index

The combination index (CI) was calculated for various combinations of EA and KAE in cancer cells. The following equation has been used (22):


CI = D1/DX1 + D2/DX2 + α D1.D2/DX1.DX2

Where:

- D1: concentration of EA in combination with KAE that conducted 50% cell killing.
- Dx1: concentration of EA alone that conducted 50% cell killing
- D2: concentration of KAE in combination with EA that conducted 50% cell killing.
- Dx2: concentration of KAE alone that conducted 50% cell killing.
- α= 0 for mutually exclusive modes of action.
- α= 1 for mutually nonexclusive modes of action.

CI values were calculated at the 50% effect level (Fa = 0.5), with α set to 1, assuming mutually non-exclusive modes of action due to the distinct but potentially overlapping biological pathways targeted by EA and KAE. Both compounds are known to modulate apoptosis regulators, oxidative stress pathways, and mitochondrial signaling, suggesting that their combination may enhance cytotoxic effects in cancer cells through complementary molecular mechanisms. This α value was selected as a reasonable assumption for compounds with partially overlapping mechanisms. We acknowledge that α was not experimentally optimized, and future studies are planned to evaluate the impact of varying α values on CI calculations. While this analysis provides initial insight into potential synergistic interactions, a more comprehensive pharmacological evaluation including assessment across multiple Fa levels, isobologram analysis, or Chou–Talalay multi-point modeling would strengthen the conclusions. Future studies are planned to extend CI calculations across a range of Fa values to more fully characterize the synergy profile.

CI Values were interpreted according to (22).

CI >1.3 Antagonism, CI = 1.3-1.1 Moderate Antagonism, CI = 1.1- 0.9 Additive effect,

CI = 0.9- 0.8 Sligh synergism, CI = 0.8 - 0.6 Moderate Synergism, CI = 0.6 - 0.4 Synergism, CI = 0.4 - 0.2 Strong synergism.

### Determination of caspase-3 activity in treated T47-D cells

To assess apoptosis in T47-D breast cancer cells, a colorimetric caspase-3 activity assay was performed following treatment with EA, KAE, and a combination of both. The T47-D cell line was selected for apoptosis assessment because it showed the highest sensitivity to the treatments in the antiproliferative assays and the concentrations of EA and KAE were selected based on their IC_50_ values determined in T47-D cells. Sub-IC_50_ doses were intentionally chosen to evaluate apoptosis induction under conditions that limit excessive cytotoxicity and allow detection of treatment-related apoptotic signaling rather than nonspecific cell death.

The T47-D cell line was removed from liquid nitrogen storage and rapidly thawed in a 37 °C water bath.

Once thawed, the cells were transferred into 75 cm² culture flasks containing 15 mL of RPMI-1640 medium. These flasks were then incubated overnight at 37 C˚ in 5% CO_2_ and 95% humidity in order to allow the formation of confluent layers. The cells were detached from the flask surface by adding 1 mL of trypsin-EDTA and PBS solution for 2–3 minutes. The cells were then washed with 5 mL of RPMI-1640 before being transferred to 15 mL sterile centrifuge tubes for centrifugation at 1000 rpm for 10 minutes at 4 °C. The obtained pellets were resuspended in 5 mL of RPMI-1640, and cell counting was performed. For seeding, cells were plated into labeled 75 cm² flasks at a density of 100,000 cells/mL. Four flasks were prepared, and the cells were incubated for 24 hours to allow them to adhere and begin proliferating. After this period, the medium was removed and replaced with fresh RPMI-1640 containing different treatments. These included EA (5 µM), KAE (7 µM), a combination of both (5 µM + 7 µM), and RPMI-1640 alone as a control. Treated flasks were incubated for an additional 48 hours.

Post-treatment, Caspase-3 activity was analyzed. All necessary reagents were brought to room temperature (25 °C) and prepared as per the kit instructions. On the assay day, media from the flasks were removed, and adherent cells were detached again using trypsin-EDTA and PBS, incubated for 2–3 minutes. The cells were washed with 5 mL RPMI-1640, transferred into sterile centrifuge tubes for centrifugation at 1000 rpm for 10 minutes at 4 °C. The supernatant was discarded, and cell pellets were resuspended in 3 mL of RPMI-1640. Cell viability and count were assessed using the trypan blue exclusion method, ensuring counts were within 1 × 10^6^ cells per sample. Required volumes were then transferred to labeled Eppendorf tubes. Cells were lysed through repeated freeze-thaw cycles and centrifuged at 2000 rpm for 20 minutes.

For the Caspase-3 assay, 40 μL of sample dilution buffer and 10 μL of each sample’s supernatant were added to wells in duplicate. The plate was sealed and incubated for 30 minutes at 37 °C. Following incubation, wells were washed five times with washing buffer, and any residual liquid was removed by tapping on absorbent paper.

Next, 50 μL of HRP-conjugate reagent was added to each well, followed by another 30- minute incubation at 37 °C and another round of five washes. For color development, 50 μL each of Chromogen Solutions A and B was added to the wells, gently mixed, and incubated for 15 minutes at 37 °C. Finally, 50 μL of stop solution was added to each well, turning the color from blue to yellow. Absorbance was then measured at 450 nm using a microplate reader. Caspase-3 activity was expressed as a fold change relative to the untreated control group. Fold increase was calculated by normalizing the absorbance values of treated samples to the mean absorbance of the control group, which was set to 1.

### Animals

All experimental procedures were approved by the Institutional Review Board (IRB), Faculty of Pharmacy, Applied Science Private University, Jordan (Approval No. 2025-PHA-18; April 10, 2025). The study was conducted in accordance with institutional guidelines for the care and use of laboratory animals and complied with ARRIVE reporting standards. The study involved female *Balb/C* mice, aged between 4 and 6 weeks and weighing approximately 23 to 25 grams each. These mice were housed individually in distinct cages under carefully regulated conditions. The mice were obtained from the animal house at Applied Science Private University. The animal facility maintained a stable environment with a temperature of around 25 °C, humidity levels between 50 and 60%, a 12-hour light and dark cycles, and continuous air circulation with free access to food and water.

Following tumor establishment and once tumors reached the predefined size range, animals were assigned to experimental groups using a random number sequence to ensure balanced baseline tumor volumes. Tumor dimensions were measured using a digital caliper, and tumor volume assessments were conducted without knowledge of group allocation to reduce measurement bias.

Animals were monitored daily for general health, body weight changes, grooming behavior, and signs of distress. Humane endpoints were predefined and included severe weight loss, impaired mobility, tumor ulceration, or persistent signs of pain or distress, at which point animals were humanely euthanized. No animals were excluded after randomization, and no unexpected deaths occurred during the experimental period. All collected data were included in the final analysis.

The number of animals per group (n = 5) was selected based on group sizes commonly reported in comparable preclinical breast cancer studies and in accordance with ethical considerations aimed at minimizing animal use while allowing detection of biologically meaningful treatment effects. At the end of the study, animals were euthanized by cervical dislocation in accordance with institutional guidelines.

### Determination of EA and KAE *in-vivo* therapeutic doses

EA *in-vivo* dose was selected to be 50 mg/kg/day to be injected intraperitoneally based on pervious study (23). Whereas, KAE *in-vivo* dose was 25 mg/kg/day to be injected intraperitoneally according to previous study (24).

### Tumor induction and measurement of antitumor activity

EMT-6/P breast cancer cells were harvested and cultured in 75cm^2^ flasks containing 15 ml of MEM. Viables cells were detached after 24 hours and washed using PBS. Cells were counted and viability was confirmed using the trypan blue assay. A suspension of 1.5 million cells/1ml of MEM was prepared and a tumorigenic dose of 150,000cells/100μl was injected subcutaneously in the bdominal area of female *Balb/C* mice. After inoculation tumors were allowed to grow for 14 days. Tumor volumes, were measured using a digital caliper and the volume was calculated using the following equation (25).


$$V\;=\;\left(L\;*\;W^{2}\right)/2$$

Where: V, L and W are the volume, length, and width of the tumor, respectively.

After tumor establishment, mice were randomly allocated into four experimental groups (n = 5 per group):

- Group I: EA group, were injected IP with 50 mg/kg/day of EA.
- Group II: KAE group, were injected IP with 25 mg/kg/day of KAE.
- Group III: Combination group, were injected IP with 50 mg/kg/day of EA and 25 mg/kg/day of KAE.
- Group IV: Control group, were injected with DMSO and PBS without any drug.

EA and KAE were freshly prepared prior to administration by dissolving each compound in DMSO and subsequently diluting with PBS to obtain the desired working concentrations. Based on mouse body weight and a fixed injection volume of 0.1 mL per mouse, EA and KAE working solutions were prepared at concentrations of 10 mg/mL and 5 mg/mL, respectively. The final concentration of DMSO in all treatment formulations did not exceed 0.1% (v/v).

Treatments were administered via IP injection at a fixed volume of 0.1 mL per mouse. Mice received the assigned treatments once daily for 10 consecutive days.

The vehicle control group received the same DMSO–PBS formulation, at an identical injection volume and dosing schedule, but without the active compounds.

Following treatment, tumor volumes were measured every other day during the 10-day treatment period. Tumor volume for each mouse was calculated using the standard formula based on caliper measurements. Tumor progression was monitored longitudinally. The percentage change in tumor volume was calculated for each mouse relative to its baseline (day 0) tumor volume using the following formula:


% Change in tumor volume = ((final tumor volume – initial tumor volume)/initial tumor volume)*100%

Negative values indicate tumor regression, whereas positive values indicate tumor progression.

Mice were classified as “tumor-free” when the tumor mass became non-palpable and remained undetectable by caliper measurement until the end of the treatment period. Tumor clearance was assessed based on external measurements only; histopathological confirmation of complete tumor eradication was not performed.

### Evaluation of kidney and liver function of treated mice

Serum samples were taken from both the control and treatment groups, and the mice underwent testing for creatinine, alanine transaminase (ALT), and aspartate transaminase (AST). According to the Commercial kits mentioned before, these were used to measure these features. A spectrophotometer with a setting at 340 nm was used to evaluate the serum levels of AST and ALT in mice after treatment with single EA, single KAE, combination and untreated mice. Additionally, the same group’s serum creatinine levels were measured, and absorbance at 500 nm was assessed.

The kit contained working reagents used to prepare samples; these were used as blanks.

For tumor volume assessment, five mice per group were included and followed throughout the treatment period. For serum biochemical analyses (ALT, AST, and creatinine), samples were collected from three mice per group. This subset was defined *a priori* in order to limit blood volume withdrawal and because three biological replicates are commonly used for exploratory biochemical evaluations. No animals were lost, excluded, or removed from the study, and no samples were missing due to technical failure. Tumor growth data were analyzed using all animals per group, whereas biochemical parameters were analyzed on a per-protocol basis using the available serum samples.

### Statistical analysis

The results are expressed as mean ± SEM (Standard Error of the Mean) as this was used to describe the reliability of the mean values obtained from independent experiments. The statistical analysis focused on comparing treatment effects between experimental groups rather than on describing the spread of individual measurements. SEM was calculated from the standard deviation using the formula SD/√n, where n denotes the number of independent experiments. For data that did not follow a normal distribution, results are additionally summarized using the median and interquartile range (IQR).

IC_50_ values for EA, KAE, and their combination across all cell lines were calculated using a non-linear regression model in SPSS (version 27, Chicago, IL). Data normality was assessed using the Shapiro–Wilk test. Homogeneity of variances was evaluated prior to parametric testing. For normally distributed data, comparisons among multiple groups were performed using one-way analysis of variance (ANOVA), followed by Tukey’s *post hoc* test for pairwise comparisons. When data did not meet the assumptions of normality, the Kruskal–Wallis test was applied, followed by Dunn’s *post hoc* test for multiple comparisons. Adjustment for multiple testing was applied within each *post hoc* procedure. Exact p-values are reported where applicable, and a p-value of less than 0.05 was considered statistically significant.

## Results

### *In vitro* results

#### Antiproliferative effects of EA and KAE as single treatments

The antiproliferative activities of EA and KAE were evaluated in EMT-6/P, MDA-MB-231, T47-D, HeLa, A549, and Vero cell lines following treatment with decreasing concentrations of each compound.

**Figure 1:**
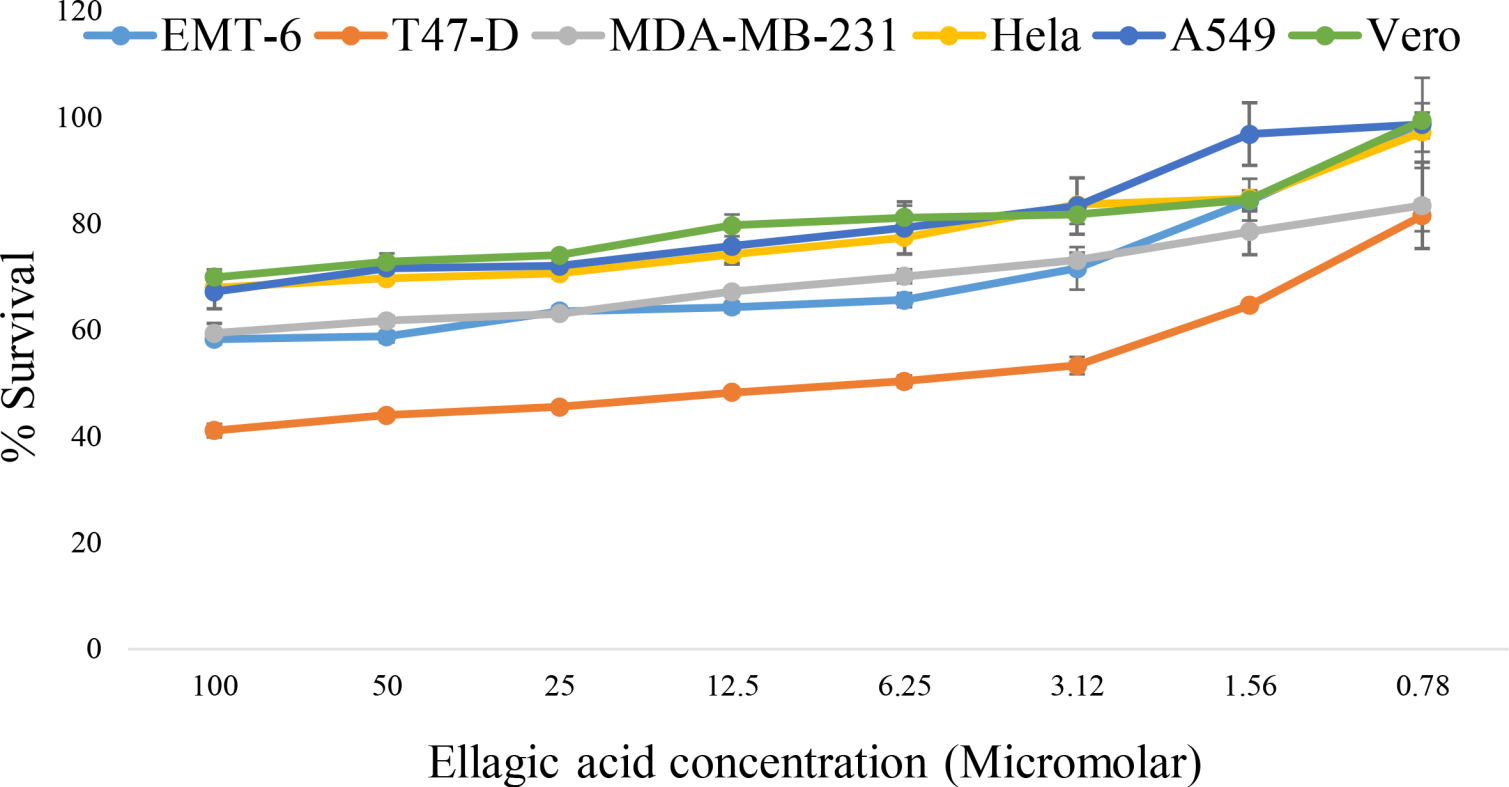
Antiproliferative effect of decreasing concentrations (µM) of EA on EMT-6/P, T47-D, MDA-MB-231, Hela, A549 and Vero cells viability.

The percentage survival as well as cell growth clearly decreased as the applied concentration of EA increased (dose-dependent manner) in EMT-6/P, T47-D, MDA-MB-231, HeLa and A549 cell lines (**Figure 1**).

**Figure 2:**
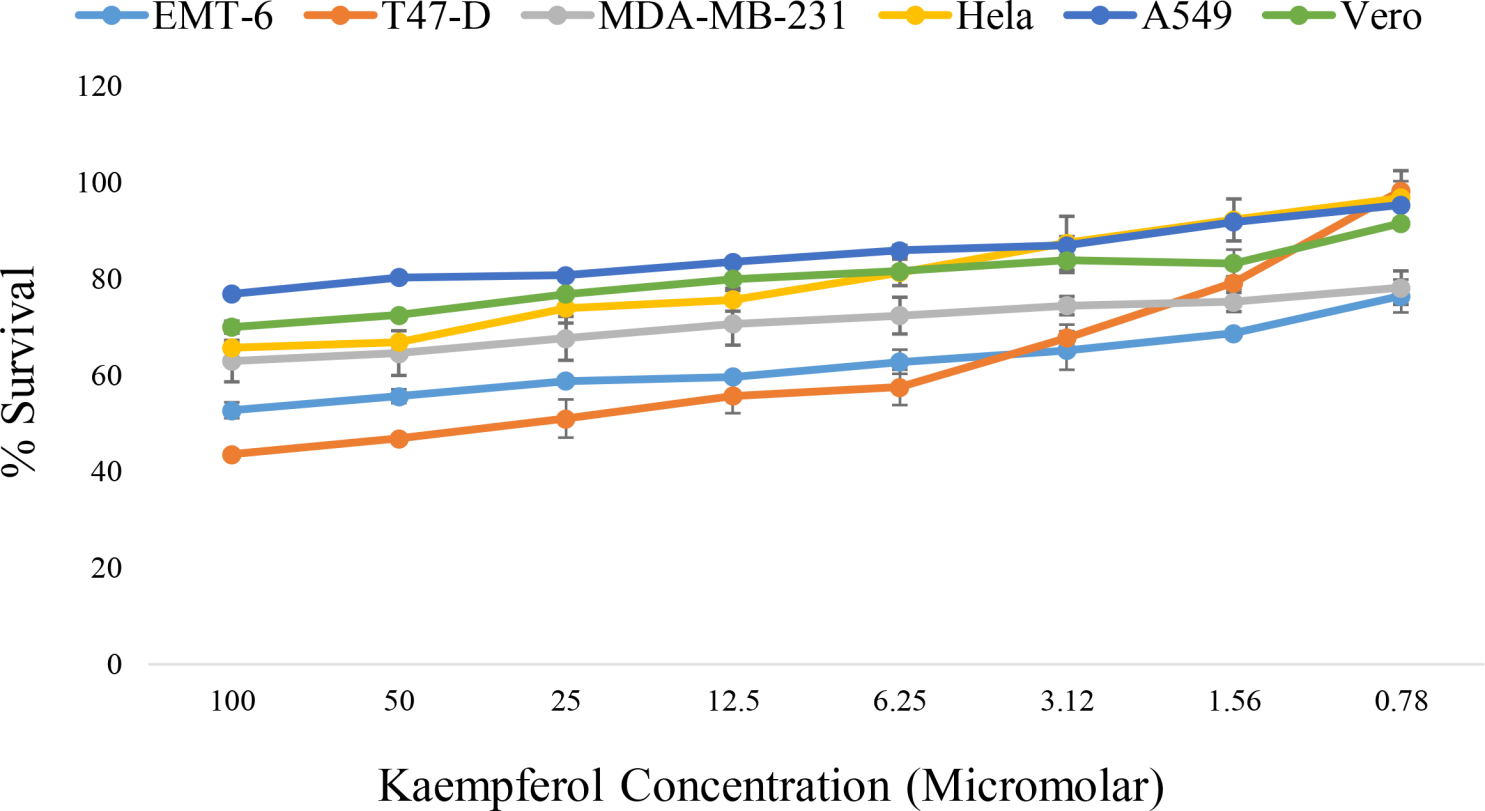
Antiproliferative effect of decreasing concentrations (µM) of KAE on EMT-6, T47-D, MDA-MB-231, Hela, A549 and Vero cells viability.

The percentage survival and cell growth decreased as the applied dose of KAE increased (dose-dependent manner) in EMT-6/P, T47-D and HeLa cell lines. However, in MDA-MB-231 and A549 cell lines, a slight decrease in inhibitory effect of KAE was detected at the highest concentration (**Figure 2**).

Single treatments with EA and KAE at their highest concentrations resulted in approximately 70% Vero cell survival (**Figures 1**, **2**).

#### Antiproliferative effects of EA and KAE in combination

To investigate the potential synergistic interaction between EA and KAE, EMT-6/P, T47-D, MDA-MB-231, HeLa, A549, and Vero cells were treated with combinations of both compounds using a fixed-concentration design, in which one agent was applied at a constant concentration while the other was tested across decreasing concentrations.

**Figure 3:**
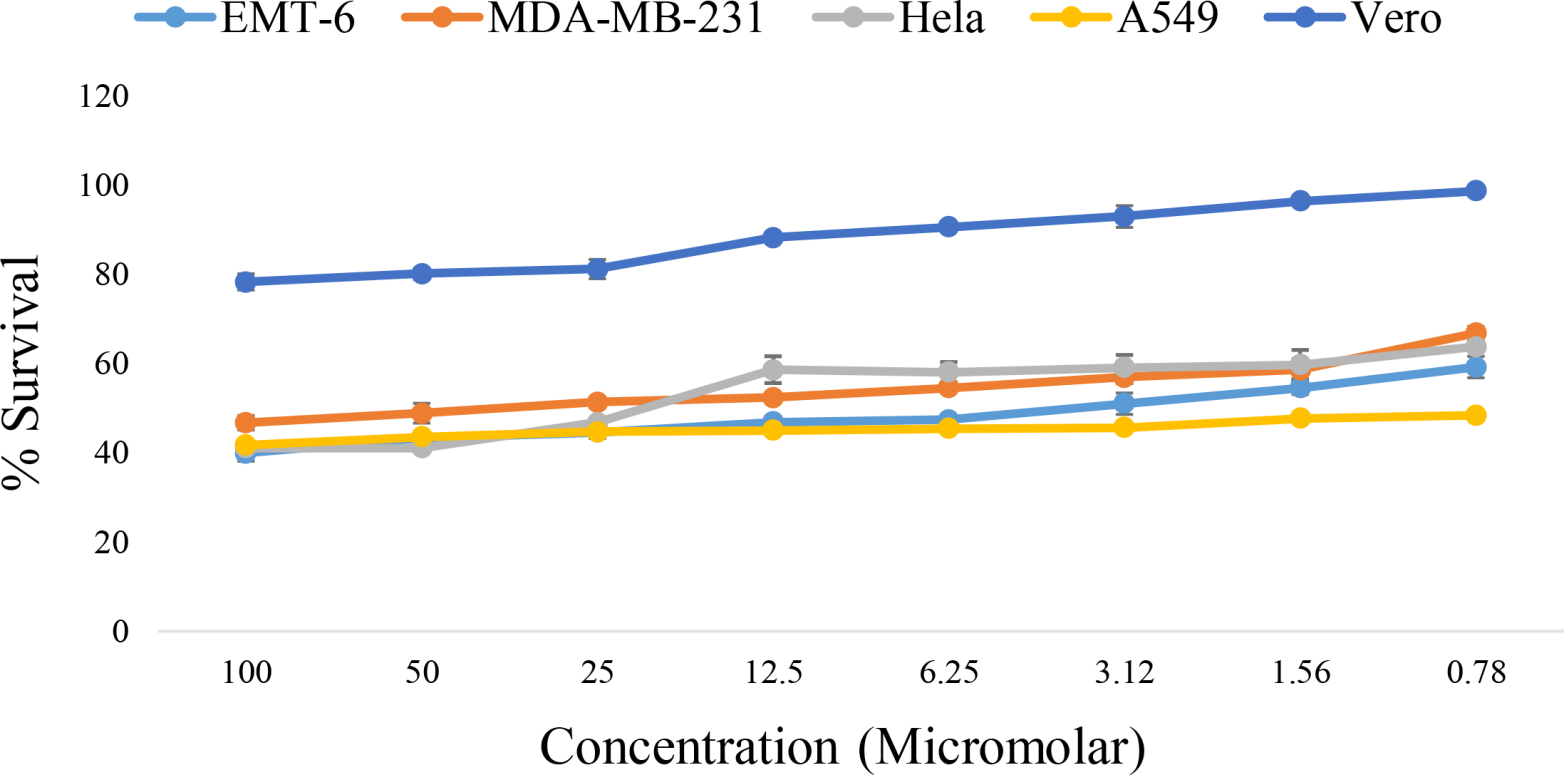
Antiproliferative effect of fixed concentration of KAE (50 µM) in combination with decreasing concentrations of EA on EMT-6/P, MDA-MB-231, Hela, A549 and Vero cells viability.

**Figure 4:**
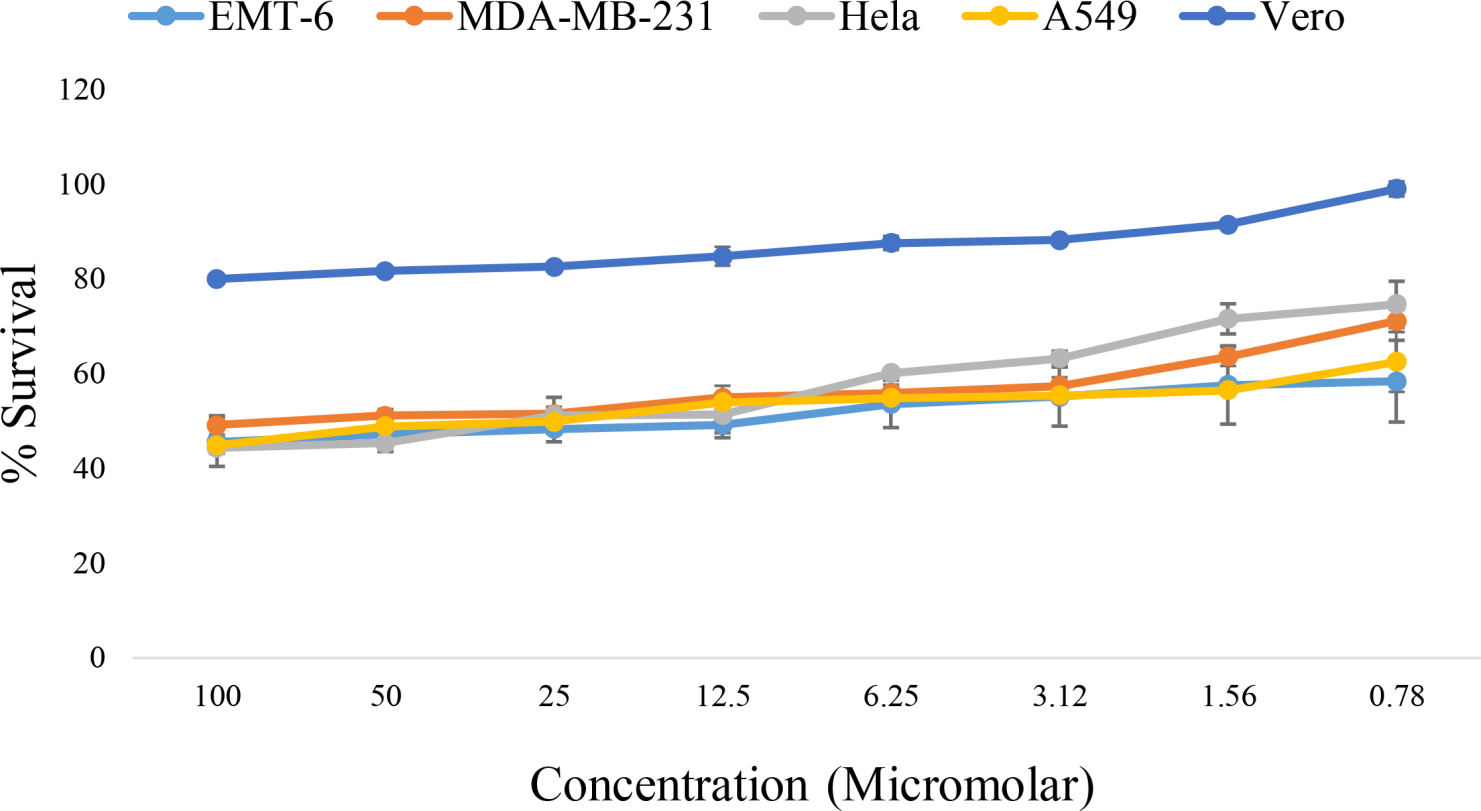
Antiproliferative effect of fixed concentration of EA (50 µM) in combination with decreasing concentrations of KAE on EMT-6/P, MDA-MB-231, Hela, A549 and Vero cells viability.

**Figure 5:**
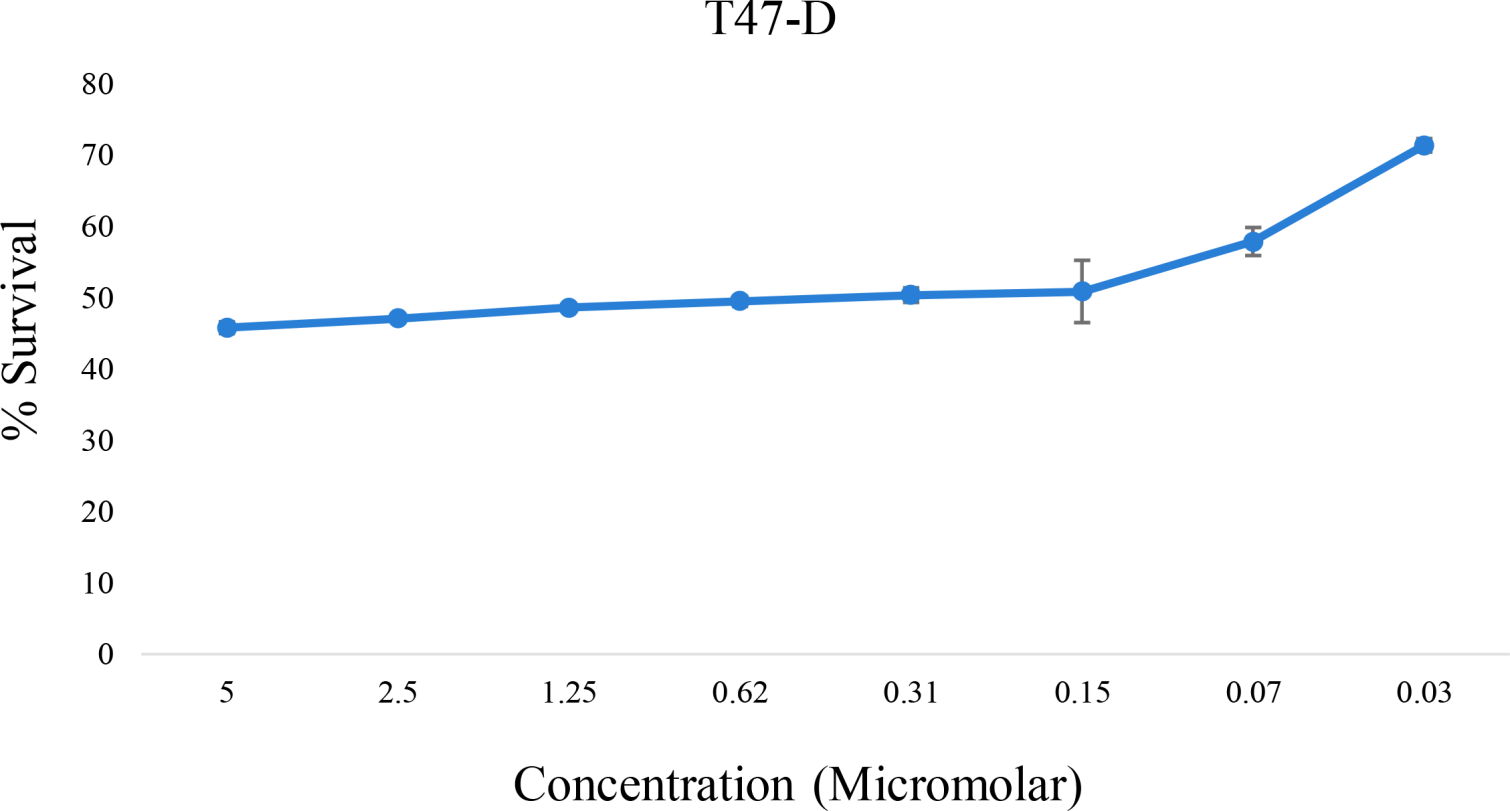
Antiproliferative effect of fixed concentration of KAE (7 µM) in combination with decreasing concentrations of EA on T47-D cells viability.

**Figure 6:**
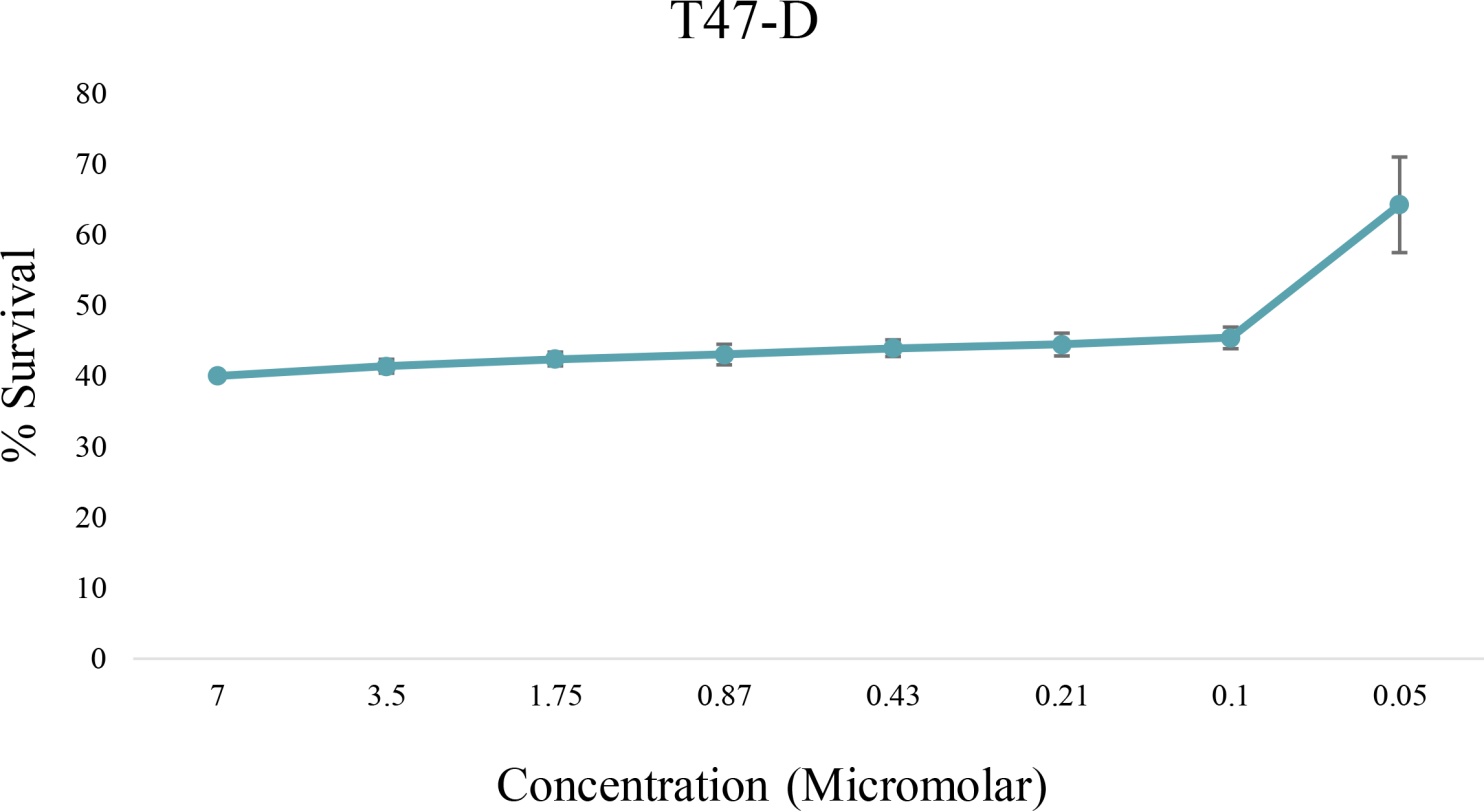
Antiproliferative effect of fixed concentration of EA (5 µM) in combination with decreasing concentrations of KAE on T47-D cells viability.

Combination treatment with EA and KAE resulted in a marked reduction in cell survival and proliferation in EMT-6/P, T47-D, MDA-MB-231, HeLa, and A549 cell lines compared with single-agent treatments (**Figures 3**-**6**).

In contrast, co-treatment with EA and KAE at their highest concentrations resulted in approximately 80% Vero cell survival (**Figures 3**, **4**).

**Table 1** summarizes IC_50_ values of EA and KAE alone and in combination, along with the calculated CI values. It is important to note that although individual IC50 values exceeded 100 µM, the combination produced IC50s within measurable ranges, confirming the validity of the synergy assessment.

**Table 1:** IC_50_ values and combination index values of EA and KAE in different cell lines:.

Cell line	EA (µM)	KAE (µM)	EA in comb (µM)	KAE in comb (µM)	CI	Interaction
Emt-6/p	>100	>100	7.4	13.8	0.22	Synergistic
T47-D	11	16	0.59	0.23	0.06	Strong synergistic
MDA-MB-231	>100	>100	20.2	22.4	0.47	Synergistic
Hela	>100	>100	23.2	18.3	0.45	Synergistic
A549	>100	>100	0.3	23.4	0.23	Synergistic
Vero	>100	>100	>100	>100	3	Antagonistic

Where: EA, Ellagic acid; KAE, Kaempferol; IC_50_, half maximal inhibitory concentration; CI, combination index. CI< 1 indicates synergism, CI = 1 indicates an additive effect, and CI > 1 indicates antagonism, µM, micro molar.

Based on CI values at Fa = 0.5, strong synergism was observed in EMT-6/P, T47-D and A549 cells, synergism in MDA-MB-231 and HeLa cells, whereas antagonism was detected in Vero cells. The observed synergistic effects in cancer cells may result from complementary modulation of molecular pathways: EA and KAE are known to induce apoptosis, alter oxidative stress balance, and affect mitochondrial signaling. This combination likely amplifies pro-apoptotic signaling and disrupts cancer cell survival pathways, leading to enhanced cytotoxicity. The antagonistic effect in normal Vero cells suggests selective targeting of cancer-specific pathways, sparing normal cell viability.

Although these results provide initial evidence of synergistic interactions, this analysis is limited to a single effect level. A comprehensive evaluation of synergy across multiple effect levels is planned for future work to confirm and extend these findings.

#### Detection of caspase-3 levels in T47-D cells

**Figure 7:**
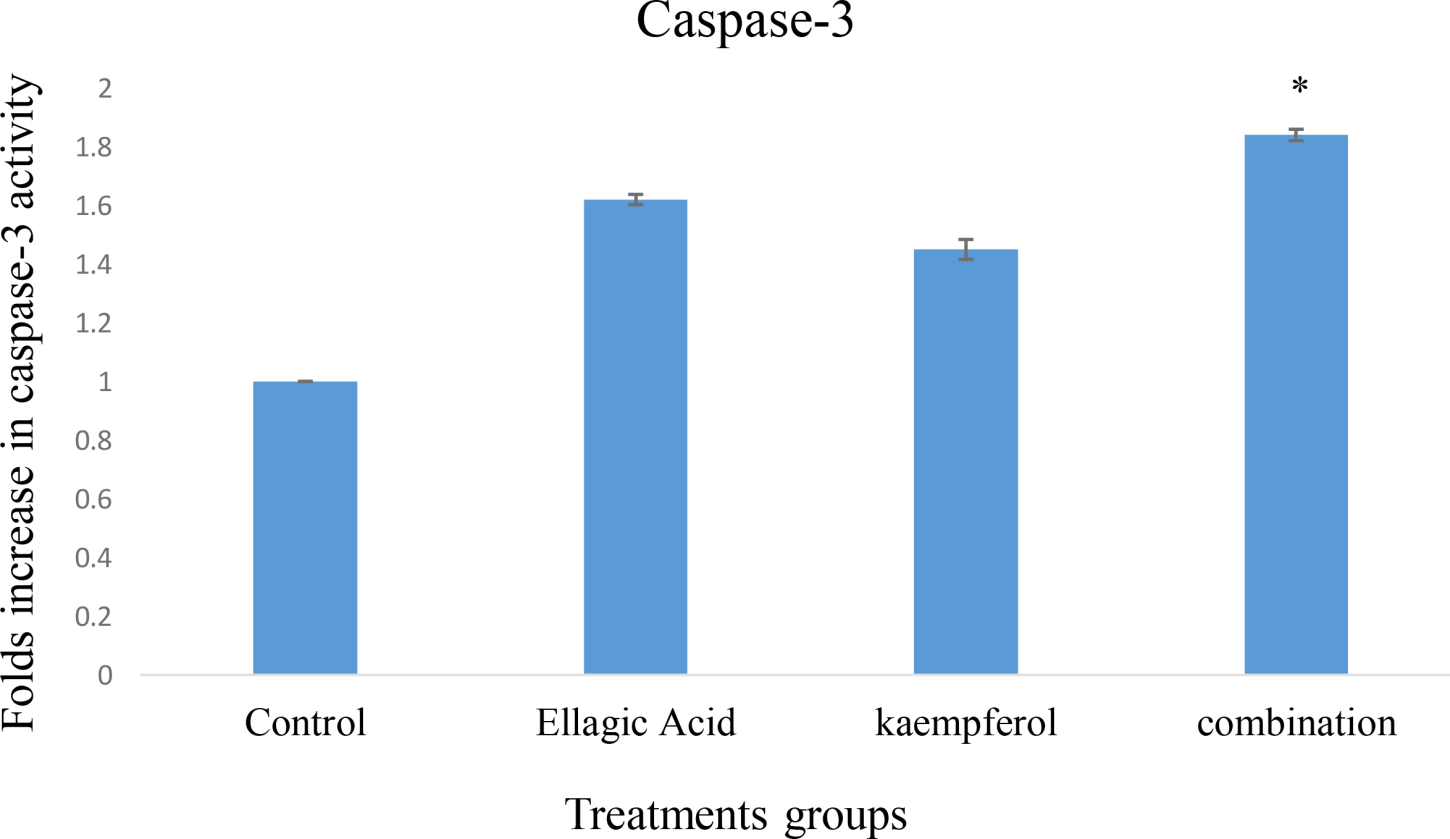
The effect of EA, KAE and their combination on caspase-3 level in T47-D cells.

To investigate the ability of the treating agents to induce apoptotic in T47-D cells, caspase-3 activity was measured after 48 hours treatment using caspase-3 assay kit. The results obtained emphasized that each of EA, KAE and their combination exerted apoptotic effect in varying degrees. The cells treated with EA and KAE combination expressed, the highest induction in caspase-3 activity, whereas cells treated with EA alone expressed lower effect but higher than that expressed by cells treated with KAE alone (p< 0.05). (**Figure 7**).

Confirming that the combination effect is greater than the sum of individual effects. This provides a mechanistic explanation at the molecular level for the observed synergy.

### *In vivo* results:

#### Antitumor activities of EA, KAE and their combination

Tumor growth was monitored throughout the 10-day treatment period. Average tumor volumes were recorded before the initiation of treatment and at the end of the study to calculate the percentage change in tumor volume for each experimental group.

**Table 2A** summarizes the effects of EA, KAE, and their combination on tumor volume in EMT-6/P tumor-bearing mice. Treatment with the EA–KAE combination resulted in the greatest reduction in tumor volume (−56.71%), followed by EA alone (−23.54%). In contrast, mice treated with KAE alone showed an increase in tumor volume (+27.46%), although this increase was markedly lower than that observed in the control group. The control group exhibited a pronounced tumor progression, with an average increase in tumor volume of 90.24%.

**Table 2A:** Effect of EA, KAE and their combination on tumor volume in EMT-6/P tumor-bearing mice after 10 days of treatment.

Group	Av. initial tumor volume (cm³)	Av. final tumor volume (cm³)	% Change in tumor volume	P - value
EA	0.296	0.226	-23.54	0.008
KAE	0.320	0.402	27.46	0.085
EA + KAE	0.327	0.137	-56.71	0.003
Control	0.382	0.730	90.24	–

Av, Average; cm³, cubic centimeter.

**Table 2B:** Treatment outcomes and tumor weight in EMT-6/P tumor-bearing mice.

Group	Tumor-free mice %	Av. tumor weight (mg)
EA	20%	234
KAE	20%	432
EA + KAE	40%	138.6
Control	0	802.4

Analysis based on median tumor volume changes yielded the same overall pattern observed with mean values, with the combination-treated group showing the most pronounced tumor regression.

**Table 2B** presents treatment outcomes in terms of tumor-free status and average tumor weight. The combination-treated group showed the highest proportion of tumor-free mice (40%) and the lowest average tumor weight. Both EA and KAE single-treatment groups demonstrated a tumor-free rate of 20%, whereas no tumor-free cases were observed in the control group.

**Figure 8:**
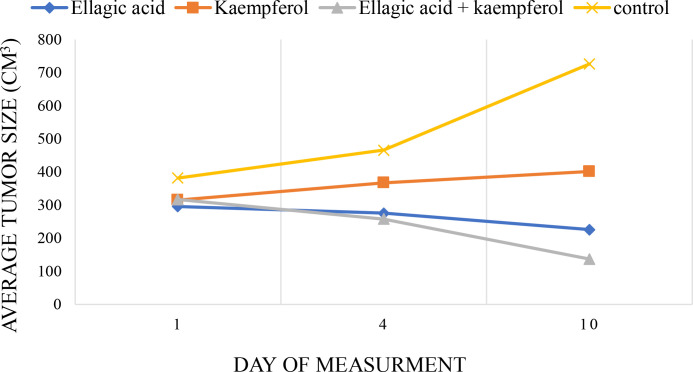
Change in average tumor volume over time during the treatment period in EMT-6/P tumor-bearing mice.

Tumor progression was monitored throughout the treatment period, and changes in average tumor volume are shown in **Figure 8**. The combination group exhibited consistently reduced tumor growth compared with the single-treatment and control groups. Individual tumor volume trajectories are not shown due to the limited number of animals per group.

Overall, combined administration of EA and KAE produced the most pronounced antitumor effect, as evidenced by tumor volume reduction, lower tumor weight, and a higher proportion of tumor-free animals. Treatment with EA alone also demonstrated measurable antitumor activity, whereas KAE alone showed limited efficacy under the conditions tested. No mortality was recorded in any treatment group during the study period.

#### Effect of EA, KAE, and their combination on serum creatinine level

**Figure 9:**
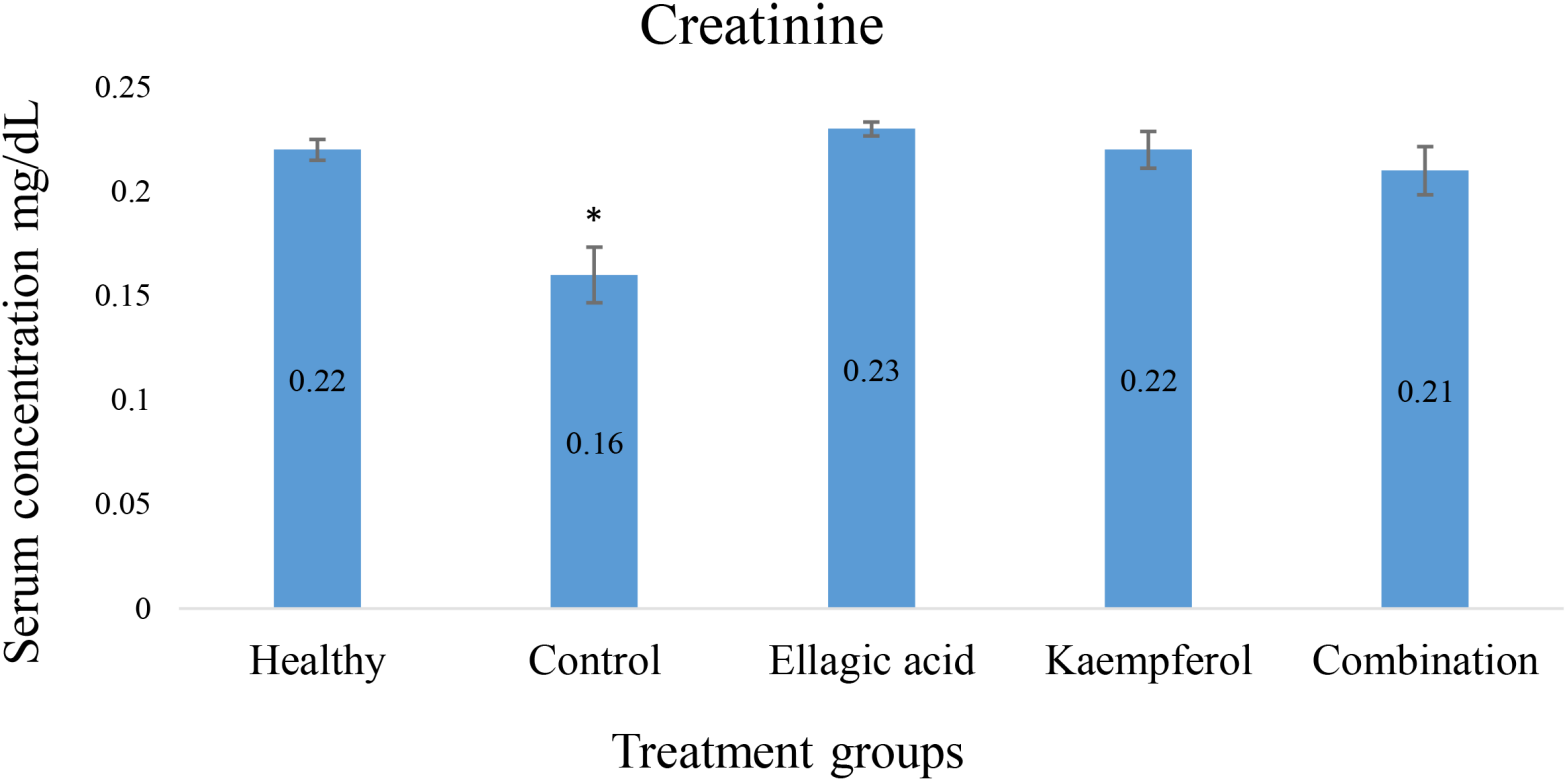
Serum creatinine levels (mg/dL, n = 3) measured in healthy mice, untreated tumor-bearing mice (control), and tumor-bearing mice treated with EA (50 mg/kg), KAE (25 mg/kg), or their combination (50 mg/kg EA + 25 mg/kg KAE).

To evaluate the potential kidney toxicity of the different treatments, serum creatinine levels were assessed. Measurements were first taken from healthy mice without tumors to establish a baseline for normal kidney function. The average serum creatinine level in these healthy mice was recorded at (0.22 ± 0.005) mg/dL. In tumor-bearing mice, serum creatinine levels varied slightly depending on the treatment administered. Mice treated with EA alone showed an average level of (0.23± 0.003) mg/dL, while those treated with KAE alone had (0.22 ± 0.008) mg/dL. The group that received a combination of both compounds exhibited a slightly lower level of (0.21 ± 0.011) mg/dL. However, the groups receiving EA alone, KAE alone, or their combination showed no statistically significant differences in serum creatinine levels compared to the healthy control group (p > 0.05), suggesting that these treatments had not detectable impact on serum creatinine levels. Notably, the untreated tumor-bearing mice (control group) exhibited a significantly lower serum creatinine level (0.16± 0.013 mg/dL) compared to the healthy group (p<0.05), indicating a potential alteration in kidney function related to tumor presence alone rather than the treatments themselves (**Figure 9**).

Where mg/dL: milligram per deciliter,

#### Liver toxicity evaluation in mice

ALT and AST are commonly used as indicators of liver toxicity, so serum levels of these liver enzymes were measured in all experimental groups. This included mice treated with EA, KAE, their combination, as well as a control group. In addition, enzyme levels were measured in healthy, tumor-free mice to provide a baseline for normal liver function.

Across all treatment groups, ALT levels were comparable to those observed in the healthy mice (45.5 ± 7.54 IU/L), the untreated tumor-bearing control group (57.43 ± 8.40 IU/L). The combination of EA and KAE resulted in the lowest ALT level, measured at (53.9 ± 15.6 IU/L). In contrast, higher ALT levels were recorded in the groups treated with EA alone (71.8± 8.6 IU/L) and KAE alone (61.7 ± 1.31 IU/L). However, these differences were not statistically significant when compared with either healthy or tumor-bearing control groups (p > 0.05).

**Figure 10:**
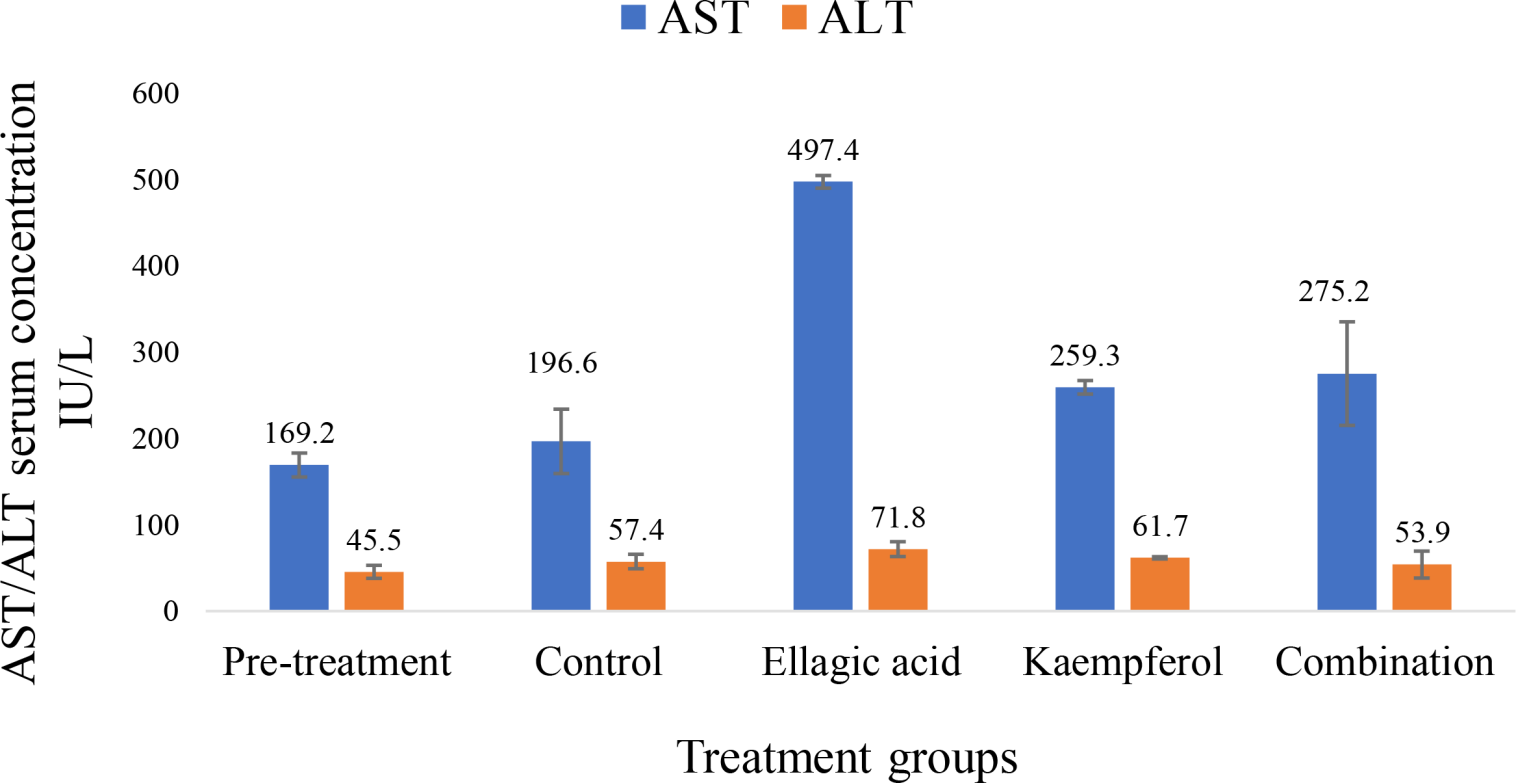
Serum ALT and AST levels (IU/L, n = 3) measured in healthy mice, untreated tumor-bearing control mice, and tumor-bearing mice treated with EA (50 mg/kg), KAE (25 mg/kg), or their combination (50 mg/kg EA + 25 mg/kg KAE). Where, IU/L, international unit per liter;, ALT, Alanine Aminotransferase; AST, Aspartate Aminotransferase.

Serum AST levels were highest in the EA-treated group (497.4 ± 7.31 IU/L), markedly exceeding those in the KAE-treated group (259.3± 7.84 IU/L), the combination group (275.2 ± 60.1 IU/L), the untreated tumor-bearing control group (196.6 ± 37.29 IU/L), and the healthy mice (169.2 ± 13.88 IU/L). Notably, AST levels in the EA group were more than double those observed in the untreated control group, and the difference was statistically significant (p< 0.05). In contrast, AST levels in mice treated with KAE alone or in combination with EA did not differ significantly from either the healthy or tumor-bearing control groups (p > 0.05) (**Figure 10**).

## Discussion

Cancer is a complex disease caused by changes in various physiological and biochemical processes. Research over recent decades shows that standard anticancer drugs can cause cancer cells to develop resistance to multiple drugs, leading to tumor recurrence. Breast cancer remains the most serious cancer-related health problem among women, affecting about 1 in 20 people worldwide and up to 1 in 8 in wealthier nations (26). More than 80% of cancer-related deaths are due to the development of MDR, prompting research into new ways to address this issue. A promising approach is to target multiple pathways in cancer cells to reduce resistance and improve treatment results while keeping side effects low. Natural substances are affordable, easy to obtain, and may provide a steady source of new agents to fight MDR through distinct biological effects. When used in combination and acting synergistically, they have the potential to improve treatment outcomes and increase patient survival rates (27).

In this study, we focused on two well-known natural agents, EA and KAE, both of which have demonstrated anticancer activity individually. However, their combined effects had not been investigated prior to this work. We evaluated the impact of EA, KAE, and their combination on various cancer cell lines, including EMT-6/P, MDA-MB-231, T47-D, HeLa, and A549, under *in vitro* conditions. Additionally, *in vivo* experiments were conducted using female *Balb/C* mice implanted with the EMT-6/P breast cancer cell line. The findings revealed that all treatment groups, particularly the combination therapy, significantly suppressed cancer cell proliferation and tumor growth in both *in vitro* and *in vivo* models. A standard chemotherapeutic agent was not incorporated into the study design because the primary focus was to assess the direct cytotoxic activity of EA and KAE, individually and in combination, and to examine their interaction. Accordingly, comparison with conventional chemotherapeutic agents was outside the intended scope of this exploratory study. The aim was to characterize the potential synergistic effects of these natural compounds rather than to perform a direct comparison with established therapies. Future studies incorporating standard-of-care agents such as doxorubicin or paclitaxel under similar experimental conditions are warranted to further evaluate relative efficacy. Based on the present findings, EA, KAE, and their combination reduced cell viability across all tested cancer cell lines. Given the consistent antiproliferative effects observed *in vitro*, an *in vivo* breast cancer mouse model was subsequently employed to further evaluate antitumor activity. In this model, treatment was associated with a reduction in tumor size and an increase in the proportion of animals exhibiting tumor regression following therapy. The *in vitro* results showed that treatment with EA alone led to a dose-dependent manner in EMT-6/P, T47-D, MDA-MB-231, HeLa, and A549 cell lines. As the concentration of EA increased, cell viability consistently declined. These results are consistent with previous studies reporting the anticancer activity of EA. In particular, EA has been shown to target CDK6 signaling in MDA-MB-231 cells, thereby suppressing cell cycle progression (28). Also in this study, EA was found to inhibit the growth of A549 lung cancer cells in a dose-dependent manner (**Figure 1**). These findings are consistent with earlier findings, which demonstrated that EA suppresses lung cancer cell proliferation by significantly reducing ATP production, disrupting mitochondrial membrane potential, and decreasing oxygen consumption under *in vitro* conditions (29).

The inhibitory effects of EA on HeLa cancer cells were examined, and the results aligned with earlier research, which reported that EA inhibits the proliferation, migration, and invasion of HeLa cells in a dose-dependent manner (30). In addition, previous research has shown that EA induces cell cycle arrest at the G1 phase and promotes apoptosis in HeLa cells (31). Collectively, these observations support the consistency of EA’s antiproliferative activity across multiple cancer cell types. In the present study, both EA and KAE exhibited their strongest antiproliferative effects against the T47-D breast cancer cell line, with IC_50_ values of 11 µM and 16 µM, respectively. In contrast, both compounds showed limited cytotoxicity toward Vero cells, with IC_50_ values exceeding 100 µM. While these findings suggest lower toxicity toward non-cancerous cells, Vero cells were used in this study as a non-cancerous comparator to provide an initial assessment of general cytotoxicity. However, as they are derived from non-human kidney tissue, they do not fully represent normal breast epithelial biology, and further studies using tissue-relevant normal breast cell models are warranted.

Single-treatment experiments with KAE demonstrated reduced proliferation in MDA-MB-231 cells, consistent with previous studies indicating that KAE induces apoptosis in MDA-MB-231 cells, mainly through a mitochondria-dependent pathway (32). The pronounced sensitivity of T47-D cells to KAE observed in this study is also in agreement with earlier findings (33).

In contrast, KAE showed a slight reduction in its inhibitory effect on A549 lung cancer cells. Regarding HeLa cells, our study showed that the survival rate decreased as the dose of KAE increased, consistent with previous findings indicating that KAE promotes apoptosis in HeLa cells through the PI3K/AKT and telomerase signaling pathways, and that its reduction of cell viability was dependent on both dose and exposure time (34). The IC_50_ values of EA and KAE when tested individually were generally greater than 100 µM, indicating low cytotoxicity of each compound alone. Interestingly, when EA and KAE were used in combination, the IC_50_ values decreased markedly compared to those observed with individual treatments. A clear dose-dependent inhibition was observed across all tested cancer cell lines following combination treatment. This reduction in IC_50_ values indicates a pronounced synergistic interaction between the two compounds. The substantial decrease in IC_50_ values in the combination groups allowed reliable calculation of combination index (CI) values. These findings suggest that the observed synergistic effect was not due to high single-agent toxicity, but rather to enhanced efficacy when both compounds were administered together. Therefore, CI calculations based on the combination IC_50_ values provide meaningful insight into the pharmacological interaction between EA and KAE.

Among the tested lines, Vero cells exhibited the highest resistance to the treatment, followed by HeLa and MDA-MB-231 cells. In contrast, T47-D cells demonstrated the greatest sensitivity, with EMT-6/P also showing notable responsiveness. Specifically, in T47-D cells, the IC_50_ values were found to be 0.59 µM for EA in combination and 0.23 µM for KAE in combination (**Table 1**).

CI values indicated a strong synergistic effect of the EA-KAE combination against EMT-6/P, A549, and T47-D cell lines, with a moderate synergistic effect observed in HeLa and MDA-MB-231 cells, and antagonism against the Vero cell line. These findings indicate that the co-administration of EA and KAE enhances anticancer activity more effectively than when either compound is used alone. Importantly, the effective concentrations required to achieve IC_50_ in combination treatments were substantially lower than those needed in single-agent applications. However, the combination experiments were performed using a fixed-dose design, in which one compound was maintained at a constant concentration while the second was tested over a concentration range. While this approach is appropriate for initial screening of drug interactions, it does not fully characterize the entire dose–response surface or interactions across multiple fixed ratios. Moreover, CI values were evaluated at a single effect level (Fa = 0.5). As a result, the observed synergistic interactions should be considered preliminary and limited to the tested conditions. More comprehensive analyses, including full matrix designs and isobologram-based evaluations across multiple effect levels, will be required to more precisely define the interaction profile between EA and KAE.

Apoptosis in the present study was assessed by measuring caspase-3 activity in a single breast cancer cell line (T47-D), using one concentration selected relative to the IC_50_ values. Both EA and KAE individually increase caspase-3 activity, indicating their ability to induce apoptosis in T47-D cells. Consistent with our findings, (35) demonstrated that ellagic acid induces DNA damage and promotes apoptosis through mitochondrial dysfunction and ROS accumulation in cancer stem-like cells, further supporting the pro-apoptotic activity of EA. These findings are further supported by a recent systematic review highlighting the consistent pro-apoptotic effects of kaempferol across diverse cancer cell lines *in vitro*, reinforcing its mechanistic role in the induction of programmed cell death (36). Interestingly, when the two compounds were used together, the increase in caspase-3 activity was even more pronounced than with either one alone. This suggests that the combination of EA and KAE may have a synergistic effect in promoting cell death through the apoptotic pathway (**Figure 7**). These findings are in line with previous studies, which also reported enhanced caspase-3 activation and apoptosis following treatment with these compounds in various cancer cell lines (37–40). Despite these observations, the apoptotic analysis in this study is limited by the use of a single cell line, a single treatment concentration, and a single biochemical readout. Moreover, histopathological confirmation of apoptotic features within tumor tissues was not performed. While caspase-3 activation provides supportive evidence of apoptosis, it does not allow a comprehensive characterization of the molecular mechanisms involved and does not fully capture the complexity or dose-dependence of apoptotic responses. Additional assays, such as Annexin V/PI staining, PARP cleavage analysis, or evaluation across multiple concentrations and cell lines, would provide a more comprehensive assessment of apoptosis and strengthen the mechanistic interpretation. These approaches should therefore be considered in future studies to further clarify the apoptotic pathways involved.

The overall findings support a synergistic interaction between EA and KAE, as reflected by CI values below 1 and reduced IC_50_ values in the combination treatment across multiple cell lines (**Table 1**). This was consistent with the increased caspase-3 activity observed in T47-D cells (**Figure 7**). However, given that apoptosis was assessed using a single marker in one cell line, these findings remain limited.

**Figure 11:**
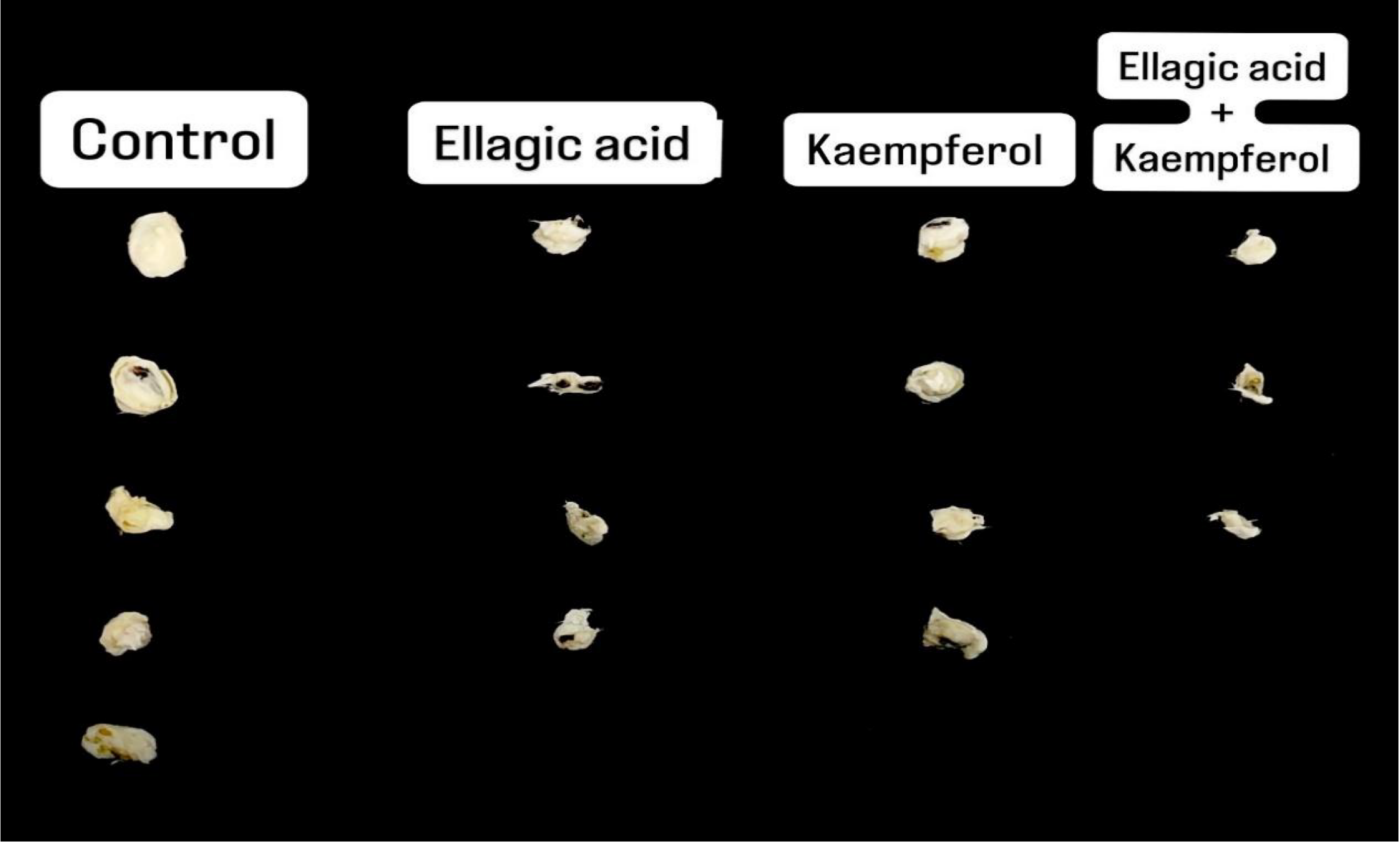
Tumor sizes in EMT-6/P after dissection at day 10 in all groups compared to each other (n=5).

The *in vivo* results show that treatment with EA at a dose of 50 mg/kg/day resulted in a 3.54% reduction in tumor size, with complete tumor regression observed in 20% of the treated mice (**Table 2**). The results are in line with earlier work showing EA’s anticancer effects *in vivo* in different tumor models. In one study, female nude mice with human MDA-MB-231 breast cancer xenografts got daily EA injections (50 or 100 mg/kg) for 25 days. Both doses reduced tumor size, the higher dose had a greater effect, pointing to a dose-dependent response. EA also reduced VEGFR-2 signaling, which suggests that it disrupts tumor vascularization. These *in vivo* results fit with earlier *in vitro* and *in silico* studies that showed EA can act directly on VEGFR-2 and block the pathways that help tumors grow and make new blood vessels (N. 23). EA has the potential to limit both tumor progression and the formation of new blood vessels, which are critical for tumor survival and metastasis (41). In contrast, KAE administered at a dose of 25 mg/kg/day did not lead to a reduction in final tumor volume when compared to the initial measurements. However, tumor progression was markedly slower compared with the control group. Specifically, tumor volume increased by 27.46% in the KAE-treated mice, whereas the control group exhibited a 90.24% increase over the same period (**Table 2**). This suggests that, at the selected dose and treatment duration, KAE exerted a tumor growth-delaying effect rather than inducing regression. Previous studies with a murine model of Ehrlich ascites carcinoma (EAC) in Swiss albino mice showed the antitumor potential of Kaempferol-3-O-α-L-rhamnoside given IP at 25 mg/kg/day and 50 mg/kg/day, that reduced viable EAC cells by about 37.2 ± 7.9% at the lower dose and 70.9 ± 6.6% at the higher dose, showing a strong dose-dependent effect, the treatment also induced apoptosis in tumor cells they used Kaempferol-3-O-α-L-rhamnoside is more water-soluble than KAE because the sugar group (α-L-rhamnose) contains polar hydroxyl (-OH) groups that interact well with water, which may enhance bioavailability. KAE without the sugar is more hydrophobic and does not dissolve easily. (24). Additional studies have also demonstrated that KAE can suppress tumor growth *in vivo* through mechanisms involving apoptosis induction and modulation of oxidative stress (F. 42). In addition, the antitumor effect of KAE was evaluated *in vivo* using Balb/C nude mice bearing bladder cancer xenografts, these mice received daily IP injections of KAE, ranging from 50 to 150 mg/kg, for 31 days, the treatment notably reduced tumor growth. (43) Taken together, these reports suggest that higher doses, improved formulation, or extended treatment periods may be required to achieve more pronounced antitumor effects with KAE alone. In our study, we evaluated the combined effect of EA (50 mg/kg/day) and KAE (25 mg/kg/day) *in vivo* to determine whether their co-administration could produce a synergistic anticancer response. To our knowledge, no previous *in vivo* studies have investigated the combined use of EA and KAE against cancer. Our results demonstrated that the combination produced a notable synergistic effect. Tumor volume was reduced by −56.71%, and complete tumor regression was observed in 40% of the treated mice (**Table 2**). Representative images of dissected tumors obtained from the different experimental groups at the end of the treatment period further support the measured differences in tumor volume and regression rates, particularly in the combination-treated group (**Figure 11**). These outcomes were significantly greater than those seen with either compound alone. This enhanced activity is in agreement with our *in vitro* findings, where combined treatment produced lower IC_50_ values and synergistic interaction profiles. All mice were randomly allocated to treatment groups following tumor establishment. Tumor measurements were performed using calipers according to a standardized protocol; however, blinding was not implemented during treatment administration or tumor measurement, which represents a limitation of the study. No treatment-related complications, behavioral changes, or unexpected mortality were observed in the treated mice during the study, indicating that the combination was well tolerated under the experimental conditions used. The enhanced antitumor activity observed with the EA–KAE combination *in vivo* was consistent with the synergistic effects identified *in vitro*. However, several limitations should be acknowledged. Histopathological evaluation of tumor tissues was not performed, and mechanistic biomarkers such as Ki-67, TUNEL, and cleaved caspase-3 were not assessed. Therefore, *in vivo* confirmation of proliferation inhibition and apoptosis induction remains limited. In addition, the intraperitoneal dosing regimen was selected based on previously published preclinical studies of similar compounds, but pharmacokinetic analysis was not conducted. Given that EA and KAE are known to exhibit limited bioavailability, the absence of pharmacokinetic profiling may affect the translational interpretation of these findings.

Overall, the combination of EA and KAE demonstrated greater antitumor activity than single-agent treatments in the present preclinical models. Nevertheless, the results should be interpreted cautiously. Future investigations should incorporate histopathological validation, mechanistic biomarker analysis, pharmacokinetic characterization, alternative dosing strategies, blinded outcome assessment, expanded dose ranges, longer treatment durations, and comprehensive toxicological evaluation to more fully define the therapeutic potential of this combination. The evaluation of toxicity and overall safety is a primary concern when investigating the therapeutic potential of any drug, especially those intended for cancer therapy (44). Since drugs are primarily metabolized and excreted by the liver and kidneys, measuring enzymes from these organs provides important clues about potential toxic effects (45). ALT and AST serum levels were measured to investigate liver function, while creatinine serum levels were used as a parameter of kidney function. Results of treated animals were compared to results obtained from normal mice that were not implanted with any tumors, as a reference for normal liver and kidney function.

In this study, we found no significant differences in serum creatinine levels between the treated and healthy mice, which suggests that kidney function was not adversely affected by EA, KAE, or their combination. This aligns with previous research showing that KAE can protect against kidney damage caused by doxorubicin without reducing its anticancer effects in breast cancer (46). Additionally protection against cyclosporine A–induced nephrotoxicity (47). Similarly, EA has been shown to mitigate cisplatin-induced renal damage in experimental models (48). Interestingly, serum creatinine levels were lower in untreated tumor-bearing mice, when compared to healthy mice (**Figure 9**). This reduction may be attributed to cancer-induced cachexia, this condition, which is frequently seen in both animal models and human patients with cancer, leads to progressive muscle loss. Since creatinine is a byproduct of muscle metabolism, diminished muscle mass results in lower creatinine production. Additionally, systemic inflammation a key feature of cachexia has also been linked to decreased serum creatinine levels (49).

Regarding liver function, the absence of significant differences in serum ALT levels across the treatment groups compared to healthy mice (**Figure 10**) suggests that the administered treatments did not exert harmful effects on liver function. While AST levels in the KAE treated and combination groups showed no significant differences from those of healthy controls, a significant elevation was detected in the EA-treated group. In the absence of a corresponding increase in ALT, this change is unlikely to indicate true hepatocellular injury. This interpretation is supported by extensive literature showing that ellagic acid does not exert hepatotoxic effects and, instead, displays hepatoprotective properties in several experimental models of liver injury, including alcohol-induced liver damage (50). It is also important to note that AST is not a liver-specific enzyme and is widely distributed in other tissues, such as the heart, skeletal muscle, kidneys, brain, and red blood cells (51). Consequently, elevations in AST may arise from non-hepatic sources. Factors including handling-related stress, muscle strain, inflammation, physical injury, or sampling-associated hemolysis could therefore have contributed to the observed increase rather than direct hepatic toxicity. In such situations, ALT, which is more specific to liver injury, often remains within the normal range (52). Consistent with this interpretation, no significant alterations in AST or ALT levels were detected in the KAE-treated group or in mice receiving the EA–KAE combination. Moreover, kaempferol has been reported to confer protective effects against hepatic injury in experimental studies (53). Taken together, these findings indicate that the combined administration of EA and KAE did not induce detectable liver or kidney toxicity under the experimental conditions used. Although statistical analyses were primarily based on p-values due to the limited sample size, the overall biochemical profile supports the conclusion that the observed antitumor effects were not accompanied by overt systemic toxicity. Nevertheless, future studies with larger cohorts, expanded biochemical panels, and histopathological confirmation will be necessary to more fully define the safety profile of these treatments.

## Conclusion

The combination of EA and KAE showed a synergistic anti-tumor effect against different cancer cell lines, including EMT-6/P, T47-D, HeLa, A549, and MDA-MB-231. Additionally, it significantly enhanced apoptosis compared to single treatments, by increasing caspase-3 activation, providing biochemical evidence of enhanced apoptotic signaling at the tested concentration.

In the animal model using *Balb/C* mice implanted with EMT-6/P breast cancer cells, the EA and KAE combination led to a reduction in tumor size and a higher proportion of tumor-free animals. Assessment of serum biochemical markers indicated no evidence of overt renal toxicity and no consistent signs of hepatotoxicity in the combination treated group under the experimental conditions used. Altogether, these findings support the potential of EA and KAE as complementary agents with enhanced anticancer activity in preclinical *in vitro* and *in vivo* models. While these early results are promising, more studies will be needed to fully understand how this combination works and how it might be used in cancer treatment.

## Data Availability

The original contributions presented in the study are included in the article/supplementary material. Further inquiries can be directed to the corresponding author.

## References

[1] Lima SM Kehm RD Terry MB . Global breast cancer incidence and mortality trends by region, age-groups, and fertility patterns. EClinicalMedicine. (2021) 38:100985. doi: 10.1016/j.eclinm.2021.100985. PMID: 34278281 PMC8271114

[2] Wang L Zhao X Fu J Xu W Yuan J . The role of tumour metabolism in cisplatin resistance. Front Mol Biosci. (2021) 8:691795. doi: 10.3389/fmolb.2021.691795. PMID: 34250022 PMC8261055

[3] Arnold M Morgan E Rumgay H Mafra A Singh D Laversanne M . Current and future burden of breast cancer: Global statistics for 2020 and 2040. Breast. (2022) 66:15–23. doi: 10.1016/j.breast.2022.08.010. PMID: 36084384 PMC9465273

[4] Hortobagyi GN de la Garza Salazar J Pritchard K Amadori D Haidinger R Hudis CA . The global breast cancer burden: variations in epidemiology and survival. Clin Breast Cancer. (2005) 6:391–401. doi: 10.3816/cbc.2005.n.043. PMID: 16381622

[5] Emran TB Shahriar A Mahmud AR Rahman T Abir MH Siddiquee MF-R . Multidrug resistance in cancer: understanding molecular mechanisms, immunoprevention and therapeutic approaches. Front Oncol. (2022) 12:891652. doi: 10.3389/fonc.2022.891652. PMID: 35814435 PMC9262248

[6] Hashem S Ali TA Akhtar S Nisar S Sageena G Ali S . Targeting cancer signaling pathways by natural products: Exploring promising anti-cancer agents. Biomedicine Pharmacotherapy. (2022) 150:113054. doi: 10.1016/j.biopha.2022.113054. PMID: 35658225

[7] Huang M Lu J-J Ding J . Natural products in cancer therapy: Past, present and future. Nat Prod Bioprospect. (2021) 11:5–13. doi: 10.1007/s13659-020-00293-7. PMID: 33389713 PMC7933288

[8] Naviglio S Della Ragione F . Naturally occurring molecules and anticancer combination therapies in the era of personalized medicine and economic crisis. Curr Pharm Des. (2013) 19:5325–6. doi: 10.2174/1381612811319300001. PMID: 23394089

[9] Newman DJ Cragg GM . Natural products as sources of new drugs from 1981 to 2014. J Nat Prod. (2016) 79:629–61. doi: 10.1021/acs.jnatprod.5b01055. PMID: 26852623

[10] García-Niño WR Ibarra-Lara L Cuevas-Magaña MY Sánchez-Mendoza A Armada E . Protective activities of ellagic acid and urolithins against kidney toxicity of environmental pollutants: A review. Environ Toxicol Pharmacol. (2022) 95:103960. doi: 10.1016/j.etap.2022.103960 35995378

[11] Tošović J Bren U . Antioxidative action of ellagic acid—A kinetic DFT study. Antioxidants. (2020) 9:587. doi: 10.3390/antiox9070587 32640518 PMC7402119

[12] Bains M Kaur J Akhtar A Kuhad A Sah SP . Anti-inflammatory effects of ellagic acid and vanillic acid against quinolinic acid-induced rat model of Huntington's disease by targeting IKK-NF-κB pathway. Eur J Pharmacol. (2022) 934:175316. doi: 10.1016/j.ejphar.2022.175316. PMID: 36209926

[13] De R Sarkar A Ghosh P Ganguly M Karmakar BC Saha DR . Antimicrobial activity of ellagic acid against Helicobacter pylori isolates from India and during infections in mice. J Antimicrob Chemother. (2018) 73:1595–603. doi: 10.1093/jac/dky079. PMID: 29566160

[14] Salinger-Martinovic S Cosic V Stojiljkovic N Ilic S Stojanovic N Dencic T . Impact of ellagic acid application on doxorubicin-induced cardiovascular toxicity model. Can J Physiol Pharmacol. (2021) 99:185–91. doi: 10.1139/cjpp-2020-0404. PMID: 33509026

[15] Gupta A Singh AK Kumar R Jamieson S Pandey AK Bishayee A . Neuroprotective potential of ellagic acid: a critical review. Adv Nutr. (2021) 12:1211–38. doi: 10.1093/advances/nmab007. PMID: 33693510 PMC8321875

[16] Chauhan A Yadav M Chauhan R Basniwal RK Pathak VM Ranjan A . Exploring the potential of ellagic acid in gastrointestinal cancer prevention: recent advances and future directions. Oncol Ther. (2024) 12:685–99. doi: 10.1007/s40487-024-00296-1. PMID: 39222186 PMC11574235

[17] Bangar SP Chaudhary V Sharma N Bansal V Ozogul F Lorenzo JM . Kaempferol: A flavonoid with wider biological activities and its applications. Crit Rev Food Sci Nutr. (2023) 63:9580–604. doi: 10.1080/10408398.2022.2067121. PMID: 35468008

[18] Imran M Salehi B Sharifi-Rad J Aslam Gondal T Saeed F Imran A . Kaempferol: A key emphasis to its anticancer potential. Molecules. (2019) 24:2277. doi: 10.3390/molecules24122277. PMID: 31248102 PMC6631472

[19] Liao W Chen L Ma X Jiao R Li X Wang Y . Protective effects of kaempferol against reactive oxygen species-induced hemolysis and its antiproliferative activity on human cancer cells. Eur J Med Chem. (2016) 114:24–32. doi: 10.1016/j.ejmech.2016.02.045. PMID: 26974372

[20] Mahmod AI Oqal M Khalid AM Afifi FU Talib WH . Phytochemical analysis, antioxidant, and antitumor activity of Ligustrum ovalifolium leaves grown in Jordan: an *in vitro* and *in vivo* study. Pharmacia. (2024) 71:1–10. doi: 10.3897/pharmacia.71.e111517

[21] Haif SK Al Kury LT Talib WH . Combination of thymoquinone and intermittent fasting as a treatment for breast cancer implanted in mice. Plants. (2023) 13:35. doi: 10.3390/plants13010035. PMID: 38202341 PMC10780740

[22] Ichite N Chougule MB Jackson T Fulzele SV Safe S Singh M . Enhancement of docetaxel anticancer activity by a novel diindolylmethane compound in human non–small cell lung cancer. Clin Cancer Res. (2009) 15:543–52. doi: 10.1158/1078-0432.ccr-08-1558. PMID: 19147759 PMC2866624

[23] Wang N Wang Z-Y Mo S-L Loo TY Wang D-M Luo H-B . Ellagic acid, a phenolic compound, exerts anti-angiogenesis effects via VEGFR-2 signaling pathway in breast cancer. Breast Cancer Res Treat. (2012) 134:943–55. doi: 10.1007/s10549-012-1977-9. PMID: 22350787 PMC3409373

[24] Akter M Parvin MS Hasan MM Rahman MAA Islam ME . Anti-tumor and antioxidant activity of kaempferol-3-O-alpha-L-rhamnoside (Afzelin) isolated from Pithecellobium dulce leaves. BMC Complementary Med Ther. (2022) 22:169. doi: 10.1186/s12906-022-03633-x. PMID: 35733130 PMC9219166

[25] Faustino-Rocha A Oliveira PA Pinho-Oliveira J Teixeira-Guedes C Soares-Maia R Da Costa RG . Estimation of rat mammary tumor volume using caliper and ultrasonography measurements. Lab Anim. (2013) 42:217–24. doi: 10.1038/laban.254. PMID: 23689461

[26] Britt KL Cuzick J Phillips K-A . Key steps for effective breast cancer prevention. Nat Rev Cancer. (2020) 20:417–36. doi: 10.1038/s41568-020-0266-x. PMID: 32528185

[27] Taylor WF Moghadam SE Moridi Farimani M N. Ebrahimi S Tabefam M Jabbarzadeh E . A multi-targeting natural compound with growth inhibitory and anti-angiogenic properties re-sensitizes chemotherapy resistant cancer. PloS One. (2019) 14:e0218125. doi: 10.1371/journal.pone.0218125. PMID: 31185048 PMC6559640

[28] Yousuf M Shamsi A Khan P Shahbaaz M AlAjmi MF Hussain A . Ellagic acid controls cell proliferation and induces apoptosis in breast cancer cells via inhibition of cyclin-dependent kinase 6. Int J Mol Sci. (2020) 21:3526. doi: 10.3390/ijms21103526. PMID: 32429317 PMC7278979

[29] Duan J Li Y Gao H Yang D He X Fang Y . Phenolic compound ellagic acid inhibits mitochondrial respiration and tumor growth in lung cancer. Food Funct. (2020) 11:6332–9. doi: 10.1039/d0fo01177k. PMID: 32608435

[30] Xia J Xue C Yu J . Ellagic acid inhibited cervical cancer growth via blocking the AKT/mTOR/STAT3 pathway. Arch Med Sci. (2020). doi: 10.5114/aoms.2020.100837

[31] Li LW Na C Tian SY Chen J Ma R Gao Y . Ellagic acid induces HeLa cell apoptosis via regulating signal transducer and activator of transcription 3 signaling. Exp Ther Med. (2018) 16:29–36. doi: 10.3892/etm.2018.6182. PMID: 29896225 PMC5995030

[32] Zhu L Xue L . Kaempferol suppresses proliferation and induces cell cycle arrest, apoptosis, and DNA damage in breast cancer cells. Oncol Res. (2019) 27:629. doi: 10.3727/096504018x15228018559434. PMID: 29739490 PMC7848404

[33] Zava DT Duwe G . Estrogenic and antiproliferative properties of genistein and other flavonoids in human breast cancer cells *in vitro*. 27(1):31–40. doi: 10.1080/01635589709514498 8970179

[34] Kashafi E Moradzadeh M Mohamadkhani A Erfanian S . Kaempferol increases apoptosis in human cervical cancer HeLa cells via PI3K/AKT and telomerase pathways. Biomedicine Pharmacotherapy. (2017) 89:573–7. doi: 10.1016/j.biopha.2017.02.061. PMID: 28258039

[35] Mandal T Shukla D Pattanayak S Barman R Ashraf R Dixit AK . Ellagic acid induces DNA damage and apoptosis in cancer stem-like cells and overcomes cisplatin resistance. ACS Omega. (2024) 9:48988–9000. doi: 10.1021/acsomega.3c08819. PMID: 39713677 PMC11656259

[36] de Morais EF de Oliveira LQR Farias Morais HGD Souto Medeiros MRD Freitas RDA Rodini CO . The anticancer potential of kaempferol: A systematic review based on *in vitro* studies. Cancers. (2024) 16:585. doi: 10.3390/cancers16030585. PMID: 38339336 PMC10854650

[37] Lee HS Cho HJ Yu R Lee KW Chun HS Park JHY . Mechanisms underlying apoptosis-inducing effects of Kaempferol in HT-29 human colon cancer cells. Int J Mol Sci. (2014) 15:2722–37. doi: 10.3390/ijms15022722. PMID: 24549175 PMC3958878

[38] Salimi A Roudkenar MH Sadeghi L Mohseni A Seydi E Pirahmadi N . Ellagic acid, a polyphenolic compound, selectively induces ROS-mediated apoptosis in cancerous B-lymphocytes of CLL patients by directly targeting mitochondria. Redox Biol. (2015) 6:461–71. doi: 10.1016/j.redox.2015.08.021. PMID: 26418626 PMC4588415

[39] Sharma N Gupta M Anand P Akhter Y Al-Dayan N Majed HA . Mechanistic insight into the autophagic and apoptotic activity of kaempferol on liver cancer cells. OncoTargets Ther. (2024) 17:579–601. doi: 10.2147/ott.s460359. PMID: 39071955 PMC11283267

[40] Zheng J Li C-F . Ellagic acid inhibits gastric cancer cells by modulating oxidative stress and inducing apoptosis. Asian Pac J Trop BioMed. (2024) 14:162–9. doi: 10.4103/apjtb.apjtb_852_23. PMID: 40932375

[41] Ceci C Tentori L Atzori MG Lacal PM Bonanno E Scimeca M . Ellagic acid inhibits bladder cancer invasiveness and *in vivo* tumor growth. Nutrients. (2016) 8:744. doi: 10.3390/nu8110744. PMID: 27879653 PMC5133127

[42] Wang F Wang L Qu C Chen L Geng Y Cheng C . Kaempferol induces ROS-dependent apoptosis in pancreatic cancer cells via TGM2-mediated Akt/mTOR signaling. BMC Cancer. (2021) 21:396. doi: 10.1186/s12885-021-08158-z. PMID: 33845796 PMC8042867

[43] Amjad E Sokouti B Asnaashari S . A systematic review of anti-cancer roles and mechanisms of kaempferol as a natural compound. Cancer Cell Int. (2022) 22:260. doi: 10.1186/s12935-022-02673-0. PMID: 35986346 PMC9392350

[44] Seeff LB . Herbal hepatotoxicity. Clinics Liver Dis. (2007) 11:577–96. doi: 10.1016/j.cld.2007.06.005. PMID: 17723921

[45] Oduola T Bello I Adeosun G Ademosun A-W Raheem G Avwioro G . Hepatotoxicity and nephrotoxicity evaluation in Wistar albino rats exposed to Morinda lucida leaf extract. North Am J Med Sci. (2010) 2:230. doi: 10.4297/najms.2010.2230 PMC3347649 22574294

[46] Wu Q Chen J Zheng X Song J Yin L Guo H . Kaempferol attenuates doxorubicin-induced renal tubular injury by inhibiting ROS/ASK1-mediated activation of the MAPK signaling pathway. Biomedicine Pharmacotherapy. (2023) 157:114087. doi: 10.1016/j.biopha.2022.114087. PMID: 36481400

[47] Zhang Y Wu Q Fu H Pang J Zhang Y Zhou H . Kaempferol attenuates cyclosporine-induced renal tubular injury via inhibiting the ROS-ASK1-MAPK pathway. Naunyn-Schmiedeberg's Arch Pharmacol. (2025) 398:3001–14. doi: 10.1007/s00210-024-03409-9. PMID: 39316086

[48] Al-Kharusi N Babiker H Al-Salam S Waly M Nemmar A Al-Lawati I . Ellagic acid protects against cisplatin-induced nephrotoxicity in rats: a dose-dependent study. Eur Rev Med Pharmacol Sci. (2013) 17:299–310. 23426532

[49] das Neves W Alves CR de Souza Borges AP de Castro G Jr . Serum creatinine as a potential biomarker of skeletal muscle atrophy in non-small cell lung cancer patients. Front Physiol. (2021) 12:625417. doi: 10.3389/fphys.2021.625417. PMID: 33912068 PMC8072336

[50] Ding Y Wang L Song J Zhou S . Protective effects of ellagic acid against tetrachloride-induced cirrhosis in mice through the inhibition of reactive oxygen species formation and angiogenesis. Exp Ther Med. (2017) 14:3375–80. doi: 10.3892/etm.2017.4966. PMID: 29042921 PMC5639323

[51] Zhao L Mehmood A Soliman MM Iftikhar A Iftikhar M Aboelenin SM . Protective effects of ellagic acid against alcoholic liver disease in mice. Front Nutr. (2021) 8:744520. doi: 10.3389/fnut.2021.744520. PMID: 34595202 PMC8478122

[52] Han J-H Kwak J-Y Lee S-S Kim H-G Jeon H Cha R-R . Markedly elevated aspartate aminotransferase from non-hepatic causes. J Clin Med. (2022) 12:310. doi: 10.3390/jcm12010310. PMID: 36615110 PMC9821092

[53] Xu T Huang S Huang Q Ming Z Wang M Li R . Kaempferol attenuates liver fibrosis by inhibiting activin receptor–like kinase 5. J Cell Mol Med. (2019) 23:6403–10. doi: 10.1111/jcmm.14528. PMID: 31273920 PMC6714241

